# Laboratory Scaled-Down Cementitious Concrete Model Used for Estimating the Bearing Capacity of a Bridge Girder Based on the Similitude Theory

**DOI:** 10.3390/ma16247559

**Published:** 2023-12-08

**Authors:** Marin Amăreanu, Ionuţ-Radu Răcănel, Ciprian Nicolae Neacşu, Daniel Dumitru Morlova

**Affiliations:** 1 Faculty of Railways, Roads and Bridges, Technical University of Civil Engineering of Bucharest, 020396 Bucharest, Romania; mirel.amareanu@gmail.com (M.A.); ciprian.neacsu@deltaacm.ro (C.N.N.); 2 S.C. MADI STAR REAL 2011 S.R.L., 023361 Bucharest, Romania; daniel.morlova@yahoo.com

**Keywords:** material behavior, laboratory testing, concrete bridge, bridge beam, similitude concept, dimensional analysis, mechanical strength

## Abstract

Bridges are structures subjected to multiple types of loads and combinations during their service life. The uncertainties linked with the materials’ behavior and manufacturing processes often necessitate the testing of produced elements on a real scale. This is particularly true for bridge concrete precast girders, which are frequently tested to predict the ultimate carrying load. Testing procedures are time-consuming, expensive in terms of both time and money, and involve a large amount of logistics and auxiliary equipment and devices. Thus, testing scaled-down models in laboratory conditions and extrapolating the obtained results with respect to the real-scale element using similitude theory has become a very common alternative method in the last decade. In this paper, experimental data regarding the efficiency of dimensional analysis computation are discussed. The proposed method involves comparing the values at which failure in bending and shear occurs for a 1:10 cementitious concrete bridge beam model with respect to the values computed for the prototype beam. Regarding the obtained results, a very small difference between the test results and the calculated values can be noticed.

## 1. Introduction

Similitude theory is one of the methods used by engineers to solve many problems, both in theory and practice. The principle of the method is not new; information suggests it originated somewhere around 1730–1800, and over time, many important scientists recognized the importance of similarity and dimensional analysis in various branches of science. The aim of using similitude is, generally speaking, to establish a relationship between small-sized models and real-scale structures or objects (usually known as prototypes), termed scaling laws, in order to predict the behavior of the prototype under different types of actions. In most cases, this linking relationship between the model and the prototype is obtained through the use of dimensional analysis [1]. The use of the method is practically unlimited, unless the studied phenomenon and the response of the prototype are not suitable to be predicted by such an approach. However, in many cases, the limitation of the method is due to the very large efforts required to derive appropriate similitude conditions between the scaled-down model and the prototype [2].

The most well-known method in dimensional analysis is the Buckingham “Π” theorem [3], introduced by Edgar Buckingham and applied between 1890 and 1900 for some studies in the field of engineering. However, the mathematical concept of the method was earlier analyzed and proposed by other scientists such as John William Strutt (Lord Rayleigh) and Joseph Louis François Bertrand.

In structural engineering, the use of similitude theory has proven to be useful and efficient, both in structural design and testing. It was used for different types of problems, such as strength and stability (buckling) [4,5,6,7,8,9], acoustic and vibrations [10,11,12], and impact and rupture. In fact, the real advantages of the method are linked with the possibility to reduce, in terms of time and money, the effort to conduct real-size model testing to estimate the conformity of the designed element and its safety in use.

Following the fast growth of computing capacity and the evolution of software used in structural engineering problems, in the last decade, many studies using similitude theory and dimensional analyses were performed.

Bridges are complex structures consisting of many structural elements connected at joints, forming intricate geometrical shapes. The dimensions of these elements are large, and for this reason, testing them on-site or in the laboratory is expensive due to the involved equipment and logistics. The testing process is also time-consuming because, in many cases, it is necessary to move the element from the site to the testing place.

In modern engineering, for common structural problems, at least in the design stage, engineers build complex finite element models to describe structural behavior. This is valid for both linear and nonlinear analyses. However, this approach can sometimes lead to errors in design by under- or overestimating the strength capacity of individual elements or the overall strength capacity. This is due to the multitude of parameters describing structural behavior. Computer modeling does not cover the uncertainties linked with complex material characteristics, such as creep, shrinkage, and cracking in the case of concrete bridges, or yielding and fatigue for steel or composite bridges.

Because of these reasons, the similitude method became a common study method for the field of bridges, involving the analysis of individual elements composing the bridge or the bridge itself, as in the case of wind tunnel analyses for aeroelastic problems. Several papers describing the use of similitude theory in the field of bridges have emerged in the last decade, primarily analyzing the dynamic behavior of bridge beams [13,14,15], but also addressing more complex problems, such as the influence of the prestressing force value on the dynamic response of bridge girders [16,17].

The use of scaled-down models has often been employed in recent developments involving structural health monitoring (SHM), especially for old masonry bridges, to validate new technique approaches, such as operational modal analysis (OMA) together with fast relaxed vector fitting (FRVF) [18,19]. Due to the difficulties arising from the “in situ” testing of old bridges, these new methods allow for the establishment of the structural response through multiple modal analyses, using a scaled known input. Changes in the dynamic response of the analyzed structure in terms of frequencies and accelerations are used as measures of damage occurrence and level. Thus, the moment of the critical state of the structure can be foreseen. The modal input is then applied to a scaled-down model to confirm the results obtained through theoretical analysis.

Another useful application of similitude theory could be the estimation of the vulnerability of bridges to seismic action using incremental dynamic analysis (IDA) curves. Testing bridges under seismic action is impossible due to the fact that earthquakes are natural phenomena. Therefore, by using artificially generated and scaled accelerograms, the dynamic response of the bridge can be estimated. Subsequently, in the laboratory, the effects can be studied on scaled-down models. The input could be the same artificially generated accelerograms reduced according to the coefficients obtained using similitude theory.

Starting from this point, the present paper provides the results obtained from a laboratory and computational study conducted on a scaled-down cementitious girder bridge to establish the ultimate failure load, both in bending and shear. The small-scale model was built in the laboratory using the same concrete mix as for a prototype precast girder, whose failure load was established by calculation. The failure force established for the prototype, scaled down according to the results from the analysis using similitude theory, shows very good agreement with the force obtained following the laboratory test. Following the imposed requirements, the four model beams were manufactured and tested to obtain the bending moment and shear force values.

## 2. Materials and Methods

### 2.1. The Similitude Theory: Modeling and Dimensional Analysis

Applying similitude theory allows one to establish certain parameters that demonstrate the equivalence between a prototype element (in this study, a concrete bridge beam) and its scaled-down model. The aim is to achieve equivalence regarding the beam’s geometry, building material properties, load types, and test diagrams.

This method is applicable when one knows the variables that govern the studied process or phenomena. The similitude criteria, using dimensional analysis, are based on E. Buckingham’s “Π theorem” [13,20,21,22,23]. According to this theorem, physical processes can be described as functions of the independent similitude criteria based on the variables that govern the phenomenon.

Thus, a process described by n variables can be expressed in the form of a homogeneous equation:

(1) $$F(x_{1},x_{2},x_{3},\ldots ,x_{n})=0$$

This can be further simplified by expressing it through a similitude criteria function containing the non-dimensional products based on the proposed variables.

(2) $$f(\Pi_{1},\Pi_{2},\Pi_{3},\ldots ,\Pi_{n-r})=0,$$

The Buckingham theorem specifies that the number of non-dimensional terms of a function is represented by the difference n − r, where n is the total number of variables and is equal to the number of fundamental quantities, a function in which the analyzed variables can be expressed, and r is the rank of the dimensional matrix.

The theorem of measurement units states that it is sufficient to restrict to using three fundamental dimensions, by means of which all quantities in mechanics may be described: length, mass, and time in the International System of Units.

The experiment presented in this paper is described by eight variables, which are shown in Table 1 along with their International System measurement units.

Replacing the proposed variables in Equation (1) results in

(3) $$F(G,P,f_{\mathrm{yk}},M,A_{s},h.b.L)=0,$$

so, there are 8 variables.

Using the data from the previous table, one can build the dimensional matrix (according to Table 2), which has, as components, the powers of the particular units with respect to the fundamental dimensions.

To establish the number of non-dimensional parameters, one must determine the rank of the dimensional matrix. The rank of a matrix is given by the order of the largest determinant different than zero.

As particular parameters, the first three variables are chosen, and the matrix is built using their exponents. For the rank of the matrix to be k = 3, the determinant of the matrix must be Δ ≠ 0 [21,23].

(4) $$A=\left(\begin{array}{ccc} 1 & 1 & 1\\ 0 & 1 & -1\\ 0 & -2 & -2 \end{array}\right),\;\det(A)=-4$$

The rank of the matrix is nonzero; thus, the number of non-dimensional parameters is 8 – 3 = 5. To establish the expressions of the five parameters, a dependent relationship between the dimensions is considered, given by a product of powers [7,16,21]:

(5) $$G^{n1}P^{n2}{f_{\mathrm{yk}}}^{n3}M^{n4}{A_{s}}^{n5}h^{n6}b^{n7}L^{n8}=\mathrm{ct}.,$$

Replacing the variables from Equation (5) with their S.I. measurement units yields

(6) $${\left[\mathrm{kg}\right]}^{n1}{\left[\mathrm{kg}\cdot m\cdot s^{-2}\right]}^{n2}{\left[\mathrm{kg}\cdot m^{-1}\cdot s^{-2}\right]}^{n3}{\left[\mathrm{kg}\cdot m^{2}\cdot s^{-2}\right]}^{n4}{\left[m^{2}\right]}^{n5}{\left[m\right]}^{n6}{\left[m\right]}^{n7}{\left[m\right]}^{n8}=\mathrm{ct}.,$$

where n1, n2, n3, n4, n5, n6, n7, and n8 are unknown powers.

Regrouping the powers by the fundamental measurement units results in

(7) $${\left[m\right]}^{n2-n3+2n4+2n5+n6+n7+n8}{\left[\mathrm{kg}\right]}^{n1+n2+n3+n4}{\left[s\right]}^{-2n2-2n3-2n4}=\mathrm{ct}.,$$

Both sides of the equation (the left side and right side, respectively) need to have the same dimensions for it to be homogeneous. Due to the fact that the left side of the equation is a non-dimensional constant, the right side should also fulfill the non-dimensional condition. A system of equations composed of the powers of each fundamental unit is

(8) $$\left\{\begin{array}{c} n2-n3+2n4+2n5+n6+n7+n8=0\\ n1+n2+n3+n4=0\\ -2n2-2n3-2n4=0 \end{array},\right.$$

Determining the primary unknowns n1, n2, and n3 as functions of the unknowns n4, n5, n6, n7, and n8 is performed using these equations:

(9) $$\left\{\begin{array}{c} n1=0\\ n2=-n4-\frac{n4}{2}-n5-\frac{n6}{2}-\frac{n7}{2}-\frac{n8}{2}\\ n3=\frac{n4}{2}+n5+\frac{n6}{2}+\frac{n7}{2}+\frac{n8}{2} \end{array},\right.$$

The solution matrix follows:

(10) $$\left(\begin{array}{ccccccccc} & n1 & n2 & n3 & n4 & n5 & n6 & n7 & n8\\ & G & P & f_{yk} & M & A_{s} & h & b & L\\ \pi_{1} & 0 & -\frac{3}{2} & \frac{1}{2} & 1 & 0 & 0 & 0 & 0\\ \pi_{2} & 0 & -1 & 1 & 0 & 1 & 0 & 0 & 0\\ \pi_{3} & 0 & -\frac{1}{2} & \frac{1}{2} & 0 & 0 & 1 & 0 & 0\\ \pi_{4} & 0 & -\frac{1}{2} & \frac{1}{2} & 0 & 0 & 0 & 1 & 0\\ \pi_{5} & 0 & -\frac{1}{2} & \frac{1}{2} & 0 & 0 & 0 & 0 & 1 \end{array}\right),$$

From the solution matrix emerge five similitude criteria written in the following manner:

(11) $$\pi_{1}=\frac{M\cdot \sqrt{f_{\mathrm{yk}}}}{P\cdot \sqrt{P}};\pi_{2}=\frac{A_{s}\cdot f_{\mathrm{yk}}}{P};\pi_{3}=\frac{h\cdot \sqrt{f_{\mathrm{yk}}}}{\sqrt{P}};\pi_{4}=\frac{b\cdot \sqrt{f_{\mathrm{yk}}}}{\sqrt{P}};\pi_{5}=\frac{L\cdot \sqrt{f_{\mathrm{yk}}}}{\sqrt{P}};$$

The criterial function becomes

(12) $$f(\frac{M\cdot \sqrt{f_{\mathrm{yk}}}}{P\cdot \sqrt{P}},\frac{A_{s}\cdot f_{\mathrm{yk}}}{P},\;\frac{h\cdot \sqrt{f_{\mathrm{yk}}}}{\sqrt{P}},\;\frac{b\cdot \sqrt{f_{\mathrm{yk}}}}{\sqrt{P}},\;\frac{L\cdot \sqrt{f_{\mathrm{yk}}}}{\sqrt{P}})\;=\;0,$$

The stage at which scaling factors for determining the input force on the prototype beams can be written is reached. The obtained criteria allow for the establishing of the scaling factor as a function of the combination between different parameters involved in the phenomena. In this case, the most suitable criterion is the one that expresses the force as a function of the reinforcement area and the characteristic resistance. Starting from the condition that the π terms are non-dimensional results in

(13) $$\frac{A_{s}\cdot f_{\mathrm{yk}}}{P}=\mathrm{ct}.\;\Rightarrow \lambda P=\lambda A_{s}\cdot \lambda f_{\mathrm{yk}},$$

The scaling factor for the input test force is equal to the product of the ratio of the reinforcement areas (prototype/model) and the ratio of the characteristic resistances of the used reinforcement (prototype/model). The similitude criteria are verified by dimensional analysis.

(14) $$\frac{A_{s}\cdot f_{\mathrm{yk}}}{P}=\frac{m^{2}\cdot \frac{N}{m^{2}}}{N}\Rightarrow dimensionsless,$$

### 2.2. Prototype Beam Characteristics

#### 2.2.1. Constructive Conditions and Technical Data

**Figure 1:**
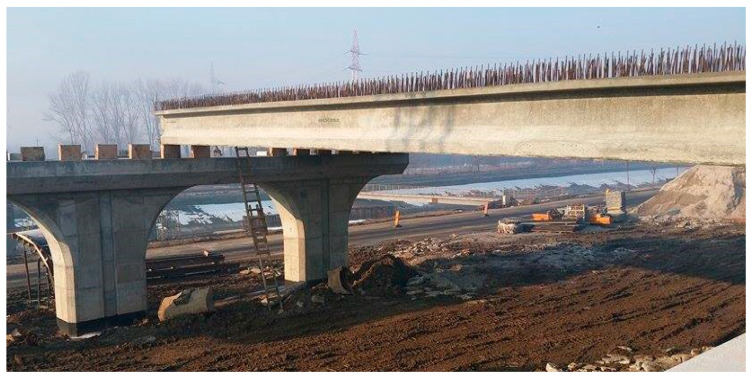
Image of the concrete beam prototype.

**Figure 2:**
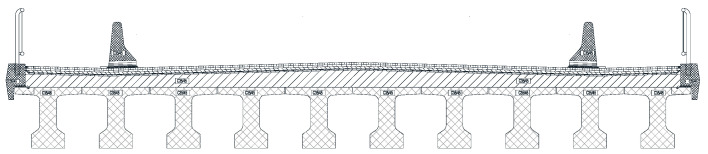
Cross section of the concrete beam.

The scaled-down model is based on a prototype concrete beam (Figure 1), forming, together with a concrete slab at the top part, a road bridge deck. There are 10 concrete beams in the deck, covering a span (Figure 2).

**Figure 3:**
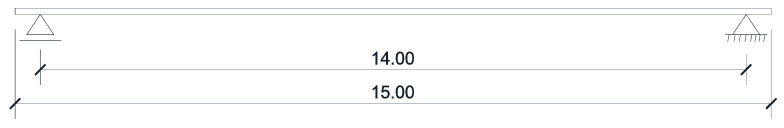
Statical scheme of the beam. Dimensions are in meters.

**Figure 4:**
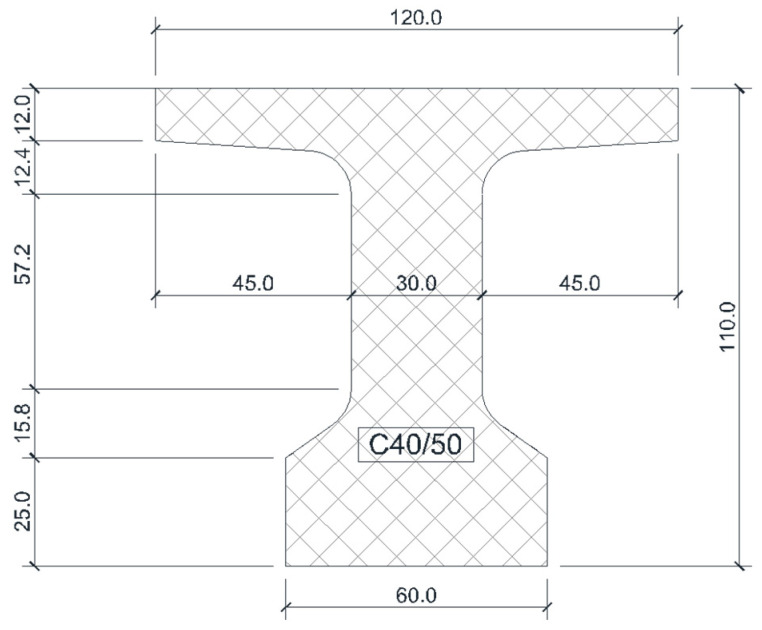
Cross section of the concrete beam. Dimensions are in cm.

The statical scheme of the analyzed concrete bridge girder is a simply supported beam (Figure 3) with a 14.00 m clear span and 15.00 m total length. The beam has a constant ‘T’-shape cross section along its entire length with a bulb and slab; the height is 1.10 m, the slab is 1.20 m wide with variable thickness, the web has a thickness of 30 cm, and the width of the bulb is 60 cm (Figure 4).

The resistance class of concrete is C40/50.

Thus results the following dimensions and technical data:

h_beam_ = 1100 mm (the height of the beam);
d’ = 100 mm (the position of the reinforcement’s center of gravity with respect to the bottom of the beam);
b_p_ = 1200 mm (the width of the slab);
h_p_ = 120 mm (the minimum height of the slab);
b = 600 mm (the width of the bulb);
d = h_beam_ − d’ = 1000 mm (the effective depth of the section);
f_cd_ = 0.85·f_ck_/1.50 = 22.66 N/mm^2^ (the design value of concrete compressive resistance);
f_pd_ = 1460 N/mm^2^ (the design value of strand resistance).

#### 2.2.2. Establishing the Loads Acting on the Beam

In order to establish the internal forces acting on the beam cross section, first, the loads acting on the entire bridge deck were considered according to EN 1990-2004, EN 1991-1-1, and EN 1991-2 [24,25,26].

The considered permanent loads were the self-weight of the bridge superstructure and the weight of the roadway layers, of the footways, and of the guardrails.

The value of the uniform distributed load produced by the permanent actions and acting of one beam was

g_perm_ = 38.62 kN/m

**Figure 5:**
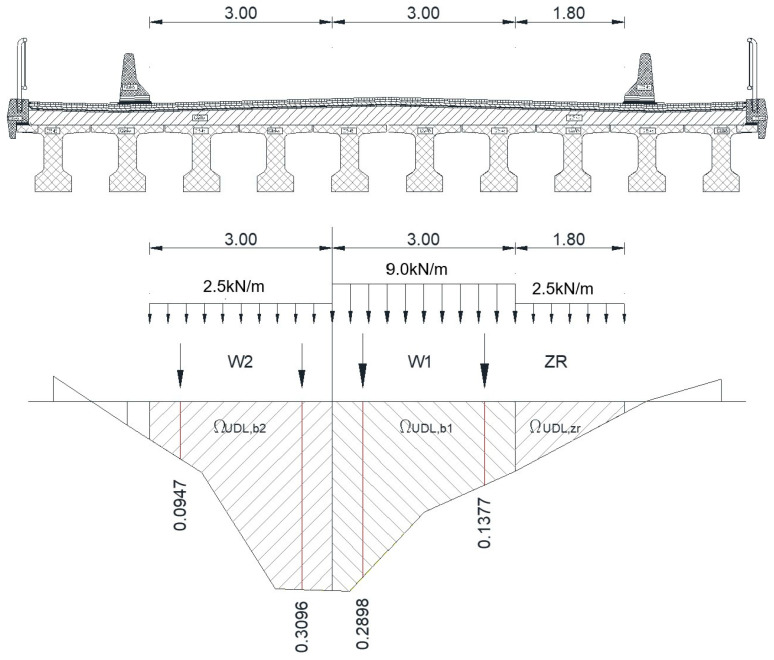
Scheme used for establishing the transverse distribution of the live loads between the beams. Length units are in meters.

For the live loads acting on the bridge, the LM1 model according to EN 1991-2 was taken into account. The permanent loads were considered evenly distributed for all beams in the bridge cross section, while the live loads were distributed according to a calculation scheme as presented in Figure 5. In this figure, W1, W2, and ZR are the width of lane 1, the width of the lane 2, and the width of the remaining zone, respectively.

In the calculation, the partial safety factors were considered with a value of 1.35 for both permanent and live loads, according to EN 1991-2.

**Figure 6:**
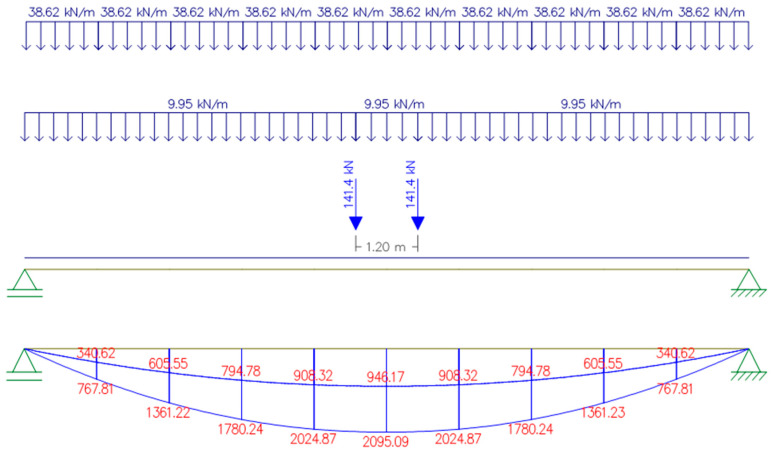
Scheme of the beam loading for maximum bending moment. In the diagram, the values are in kNm.

**Figure 7:**
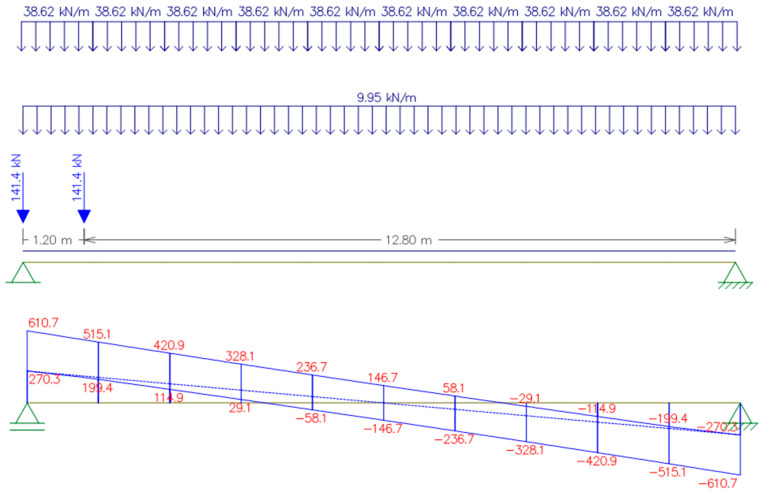
Scheme of the beam loading for maximum shear force. In the diagram, the values are in kN.

The position of the loads acting on the concrete beam and the envelope diagrams of bending and shear are shown in Figure 6 and Figure 7.

The resulting values of the beam internal forces were as follows:

*M_Ed_* = 2095.09 kNm;
*V_Ed_* = 610.70 kN.

#### 2.2.3. Designing the Prototype’s Longitudinal and Shear Reinforcement

As mentioned before, the concrete class in the beam was C40/50. The type of strand chosen was TBP15.

**Figure 8:**
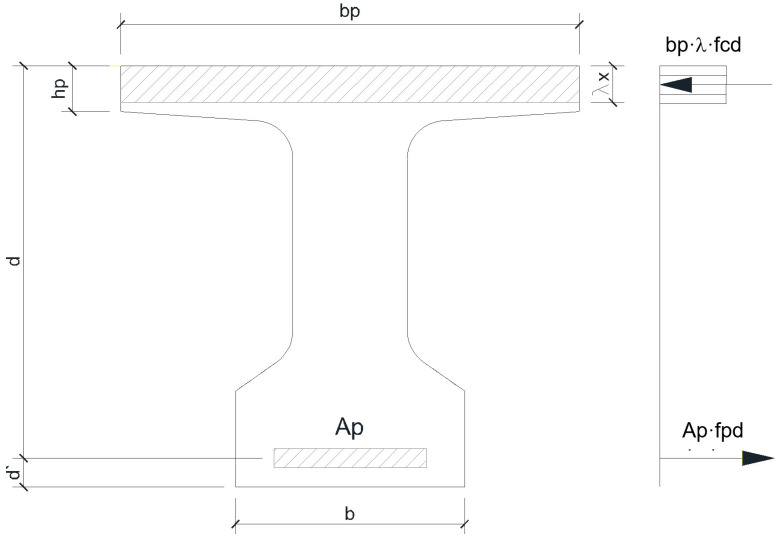
Internal forces equilibrium scheme.

To establish the necessary number of strands, equilibrium Equations (15) and (16) on the beam cross section (Figure 8) are written:

(15) $$\sum F_{X}=0\Rightarrow A_{p}\cdot f_{\mathrm{pd}}=b_{p}\cdot \lambda \cdot x\cdot f_{\mathrm{cd}},$$

(16) $$\sum M=0\Rightarrow M\;=b_{p}\cdot \lambda \cdot x\cdot f_{\mathrm{cd}}\cdot \left(d-\frac{\lambda \cdot x}{2}\right),$$

In the above equations, λ = 0.85, A_p_ is the total strands area, and x is the height of the concrete compressive zone. The other terms in both equations were defined previously.

By inputting all known data in Equation (16), the value of x (the height of the concrete compressive zone) resulted in 80.20 mm. By introducing the value of x in Equation (15), it was found that the strands area A_p_ had a value of 1494.97 mm^2^. Subsequently, considering the effective area of one strand of 139 mm^2^, the searched necessary number of strands was n_p_ = 12 TBP15.

**Figure 9:**
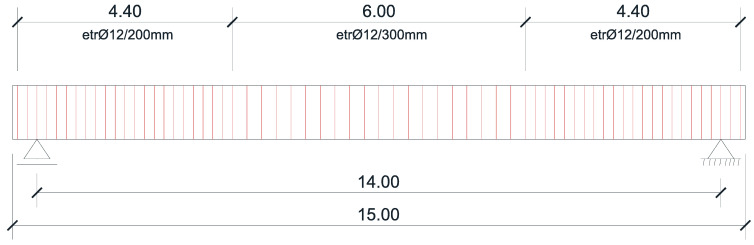
Proposed distribution of stirrups along the beam.

For the shear force reinforcement, a distribution of stirrups along the beam was proposed, as shown in Figure 9 (with the stirrups spaced at 20 mm), and the corresponding shear resistance of the cross section was checked.

Stirrups having a diameter of 12 mm and made of BST500 steel were considered.

Based on this, the shear resistance of the prototype beam is given by Equation (17):

(17) $$V_{Rd}=V_{Rd,c}+V_{Rd,s},$$

where *V_Rd_* is the shear capacity of the beam, *V_Rd,c_* is the design force resistance of the beam without stirrups, and *V_Rd,s_* is the design shear force sustained by the yielding stirrups.

Considering the proposed distribution of the stirrups resulted in

*V_Rd_* = 97 + 717.52 = 814.52 kN, and thus *V_Rd_* > *V_Ed_* = 610.70 kN.

With this, one can conclude that the necessary reinforcement of the prototype beam in bending and in shear was found.

#### 2.2.4. Characteristics of the Scaled-Down Model: Material Characteristics

The materials used for the current experiment were chosen so that their physical–mechanical properties were as close as possible to the properties of the materials used for the prototype beams.

**Figure 10:**
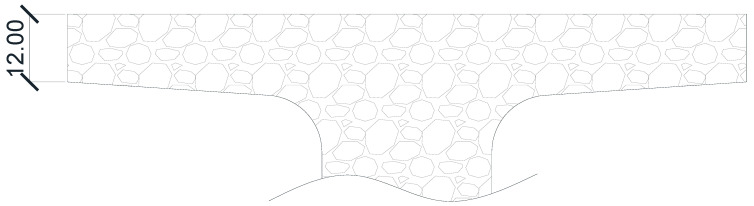
Repartition of the aggregates with respect to the beam’s slab. Dimensions are in mm.

The type and dimensions of the aggregates used for the micro-concrete mixture should meet specific requirements. For slab-type concrete elements, the maximum admissible aggregate diameter is 1/3 of the slab’s height. From Figure 10, the maximum admissible aggregate dimensions for the concrete deck are

(18) $$d_{g}=\frac{12\;\mathrm{mm}}{3}=4\;\mathrm{mm},$$

The value of the small diameter of the reinforcement (the diameter and spacing between bars are reduced based on geometrical criteria) was determined to ensure the bond with the concrete. For longitudinal reinforcement, threaded bars were used.

**Figure 11:**
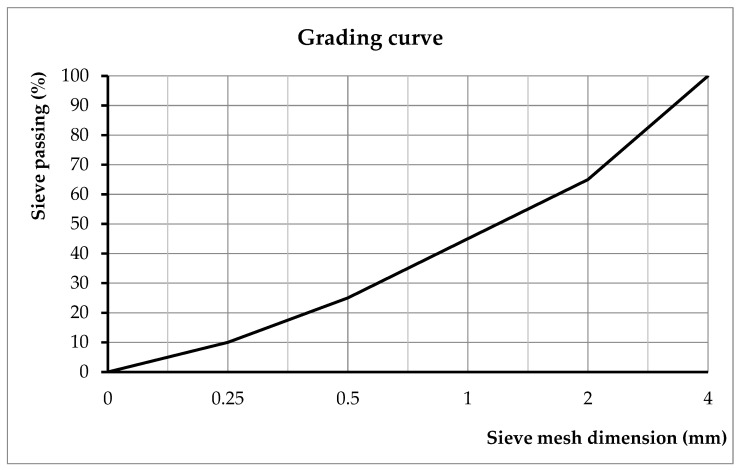
Grading curve of the concrete (experimentally obtained).

Starting from the design concrete class of the prototype, a C40/50 concrete class was chosen, with river aggregates 0–4 mm in diameter. To obtain the C40/50 concrete class according to EN 206 [27], the following conditions were taken into account:

- Water–cement ratio = 0.44;
- Water: 242 l (including the additives);
- Cement quantity: 550 kg/m^3^;
- Aggregate quantity: 1656 kg;
- A quantity of 3% from the cement quantity of superplasticization additive Sika Plast 302 S was added;
- Entrained air 2%;
- The type of cement used was CEM I 42,5 R (supplied by CARPATCEMENT Romania);
- The grading curve (Figure 11) was chosen by extrapolating the limit of the granularity zone for the 0–4 mm aggregates.

To prepare one cubic meter of concrete (1 m^3^), the following material quantities are necessary (Table 3):

To attain the characteristic concrete class, the average compressive strength of three concrete cubes with 15 cm sides (Table 4) was determined, and the class C40/50 was confirmed.

The reinforcement of the beam was also determined by similitude criteria, satisfying important parameters, such as the spacing of the stirrups along the entire length of the beam, the diameter of the stirrups, the longitudinal reinforcement area, and the use of the longitudinal distribution reinforcement to obtain a higher stiffness for the reinforcement cage.

The limitation of the concrete beam’s cross section and of the reinforcement areas A_p_ (longitudinal prestressing reinforcement) and A_sw_ (transversal reinforcement area) impose a maximum of 4 mm in diameter, used in the experiment. The characteristics of the longitudinal reinforcement’s surface must be chosen in order to ensure a correct bond with the concrete.

The longitudinal reinforcement’s tensile resistance did not represent a problem, because the scaling factor for forces was chosen, taking into account also the failure resistance of the reinforcement.

**Figure 12:**
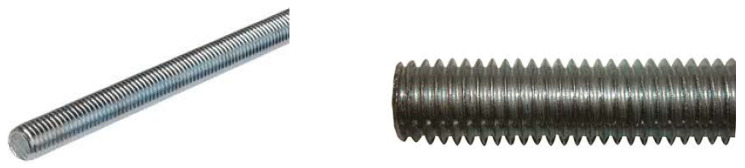
Threaded bar profile.

Because, in construction, domain bars of diameters smaller than 4 mm do not exist, threaded steel bars (rods) (Figure 12) for the simulation of the longitudinal reinforcement were used.

**Figure 13:**
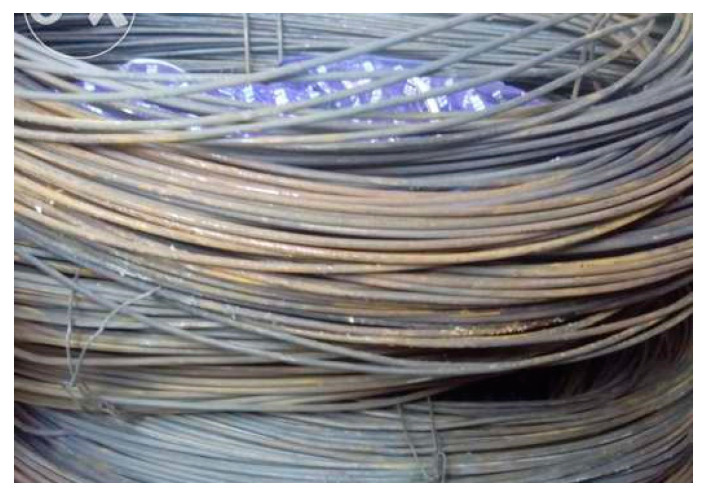
Smooth profiled wire.

The stirrups were manufactured from smooth profiled steel wires (Figure 13). Geometric similitude was also used for establishing the stirrups’ diameter in the following manner: starting from a 12 mm stirrup diameter, Ø_str_ = 12 mm.

(19) $$\phi_{\mathrm{str},\mathrm{model}}=\frac{\phi_{\mathrm{str}}}{\lambda_{L}}=\frac{12}{10}=1.20\;\mathrm{mm},$$

where *λ*_L_ = 10 is the scaling factor for length.

To maintain the reinforcement’s designed position, longitudinal repartition bars were used: 3 mm diameter wires, in this case (Figure 13).

To establish the physical–mechanical characteristics of the reinforcement, traction tests were performed on the following:

- A total of 2 threaded bars, 50 cm in length and with an effective diameter of 3.80 mm;
- A total of 2 smooth profiled bars, 50 cm in length and 1.18 mm in diameter.

**Figure 14:**
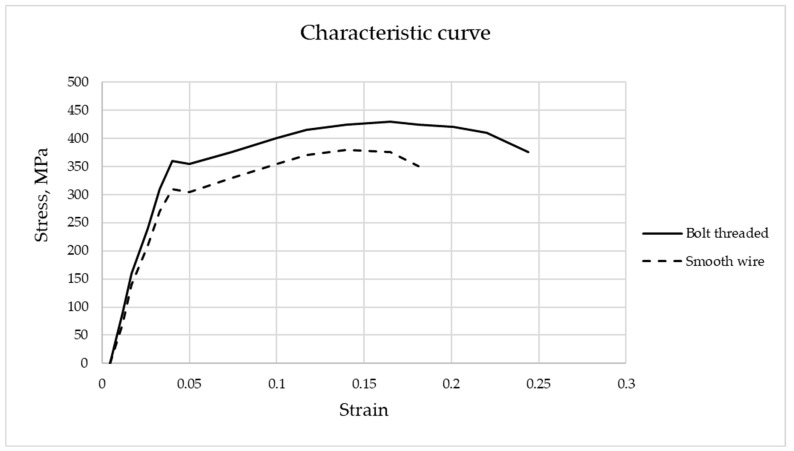
Stress–strain diagram/characteristic curve of the steels used for the reinforcement.

In Figure 14, the stress–strain curve for the two types of reinforcement is shown, and the following characteristic resistances can be deduced (Table 5).

### 2.3. Design Computation (Lengths, Spacing of the Stirrups, Bending Moment, and Shear Force) According to the Similitude Theorem: Manufacturing the Model Beams

The geometry of the models was obtained by applying the length scaling factor for all the beam’s dimensions, so the total length of the beam was

(20) $$L_{\mathrm{beam},\;\mathrm{model}}=\frac{L_{\mathrm{beam},\;\mathrm{prototype}}}{\lambda_{L}}=\frac{15}{10}=1.50\;m,$$

**Figure 15:**
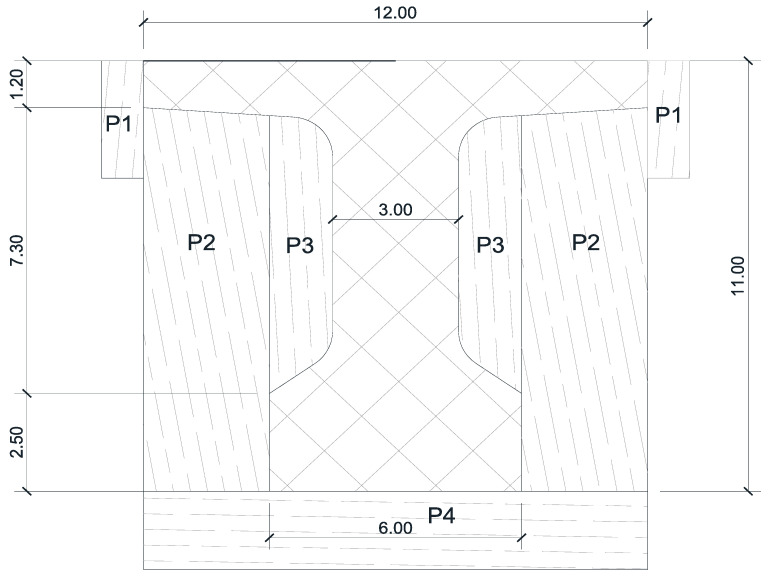
Cross section. Formwork plan. Dimensions are in cm.

The transversal section was drawn at a 1:10 scale, and a formwork plan was created (Figure 15).

**Figure 16:**
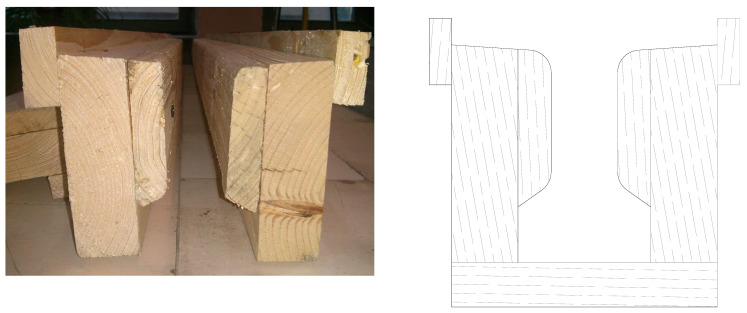
Actual formwork and sketch.

The formwork (Figure 16) was built from naturally dried (for 3 years) firewood planks. To achieve high precision and obtain smooth surfaces, the wood was planed with a surfacing machine.

**Figure 17:**
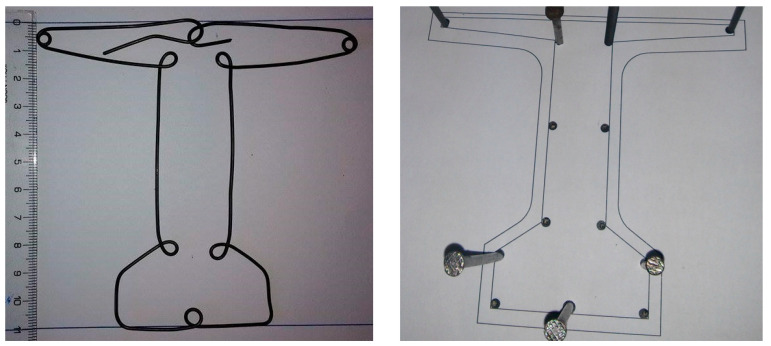
Actual obtained stirrup and used bending form.

The reinforcement cages were constructed using the prototype beam’s reinforcement plans (1.2 mm diameter steel wire, as shown in Figure 17), starting from the reduced (1:10) beam cross section.

**Figure 18:**
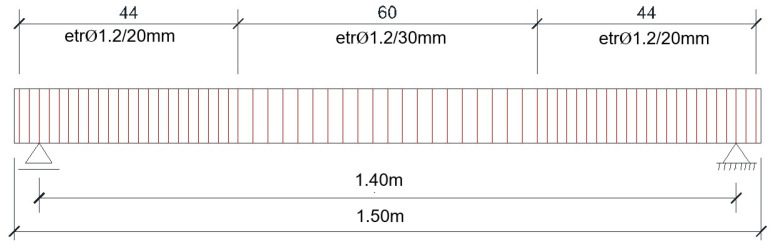
Elevation of the stirrups’ disposal.

The stirrups were arranged around the longitudinal bars, considering the distances between them. The spacing between the stirrups, as per Equation (21), resulted from the prototype’s reinforcement plan, as shown in Figure 18:

(21) $$s_{\mathrm{beam},\;\mathrm{model}}=\frac{s_{\mathrm{beam},\;\mathrm{prototype}}}{\lambda_{L}}=\frac{s\;(\mathrm{mm})}{10},$$

**Figure 19:**
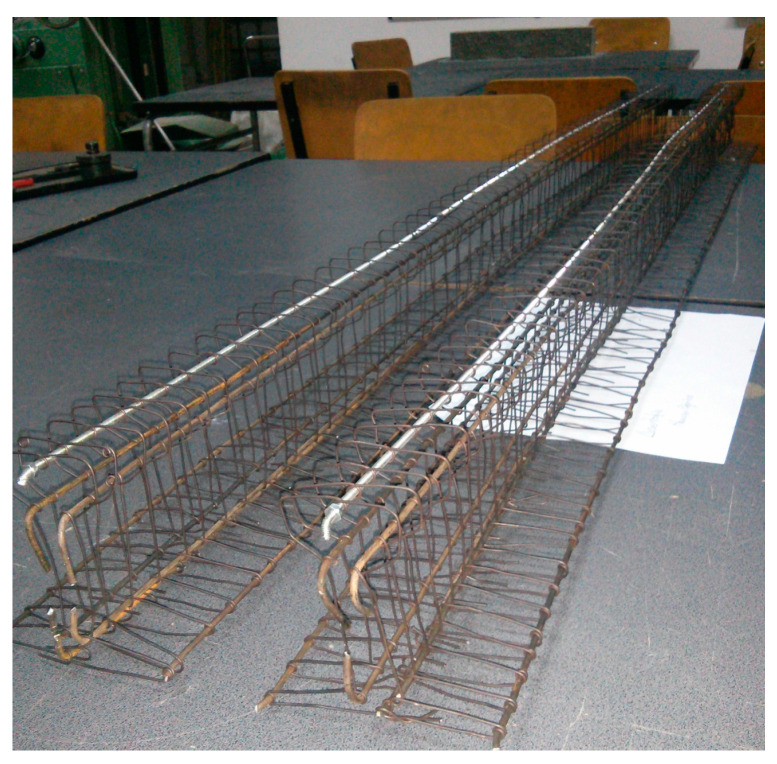
Reinforcement cages.

In Figure 19, one can observe the obtained reinforcement cages.

**Figure 20:**
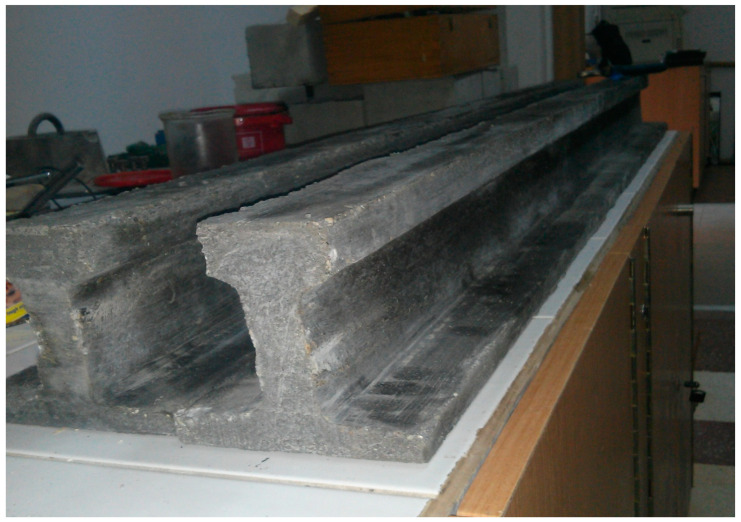
Scaled-down model beams.

A batch of concrete sufficient for two scaled-down beam models was prepared all at once (Figure 20). For the experiment, four scaled-down models were produced: two for testing in bending and two for testing in shear.

**Figure 21:**
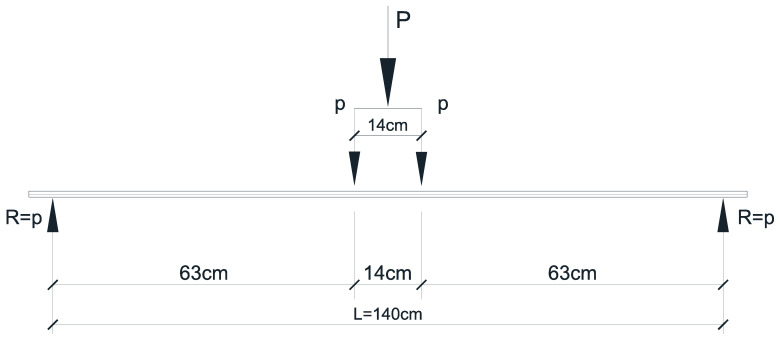
Bending test scheme.

The bending moment test is performed by applying two concentrated forces placed symmetrically with respect to half of the span (Figure 21) to obtain pure bending.

The necessary applied force P is computed as a function of the statical scheme and the maximum computed bending moment of the prototype beam (*M_Ed_*).

According to the above scheme, the prototype’s bending failure force resulted in

(22) $$p=\frac{20\cdot M_{Ed}}{9\cdot L},$$

and the total applied force was

P_prototype,M_ = 2·*p* = 665.08 kN, (23)

Then, computing the scaling factor for the input force, as in Equation (13),

(24) $$\lambda P=\lambda A_{s}\cdot \lambda f_{yk},$$

(25) $${\lambda P}_{M}=\frac{A_{p,prototype}}{A_{s,model}}\cdot \frac{f_{pd,\;protottype}}{f_{yd,model}}=280.87,$$

Thus, the value of the applied force for the reinforced concrete scaled-down model beam in bending was

(26) $$P_{\mathrm{model},M}=\frac{P_{\mathrm{prototype},M}}{{\lambda P}_{M}}=2.37\;\mathrm{kN},$$

**Figure 22:**
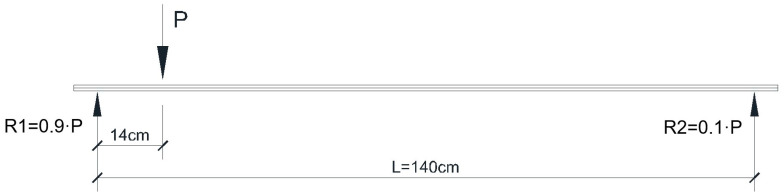
Shear test scheme.

The shear test is performed by applying a concentrated force at a distance equal to 0.1·L with respect to the beam end (Figure 22).

The necessary applied force P is computed, for this case, as a function of the maximum shear force (*V_Ed_*) and the statical scheme. It resulted in

(27) $$P_{\mathrm{prototype},V}=\frac{V_{\mathrm{Ed}}}{0.9}=678.56\;\mathrm{kN},$$

And applying again the scaling factor for the input force resulted in

(28) $${\lambda P}_{V}=\frac{A_{sw,prototype}}{A_{sw,model}}\cdot \frac{f_{pd,\;protottype}}{f_{yd,model}}=167,$$

Thus, the value of the applied force for the reinforced concrete scaled-down model beam in shear was

(29) $$P_{\mathrm{model},V}=\frac{P_{\mathrm{prototype},V}}{{\lambda P}_{V}}=4.06\;\mathrm{kN},$$

## 3. Results

The aim of this study was to establish the failure force of a 14 m prototype bridge concrete beam by using scaled-down models, the similitude theory, and dimensional analysis. Four scaled-down models were prepared and tested in the laboratory: model 1 and 2 in bending and model 3 and 4 in shear.

**Figure 23:**
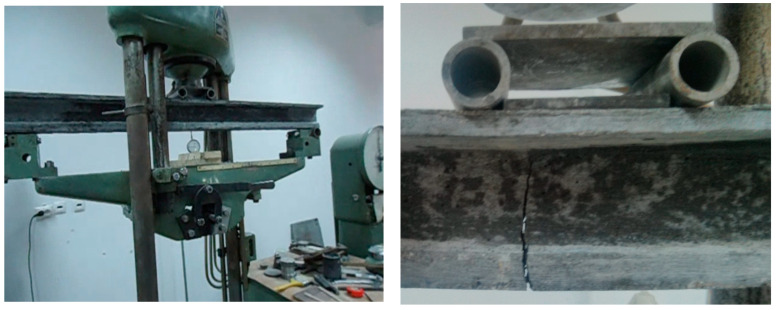
Model 1, bending test.

**Figure 24:**
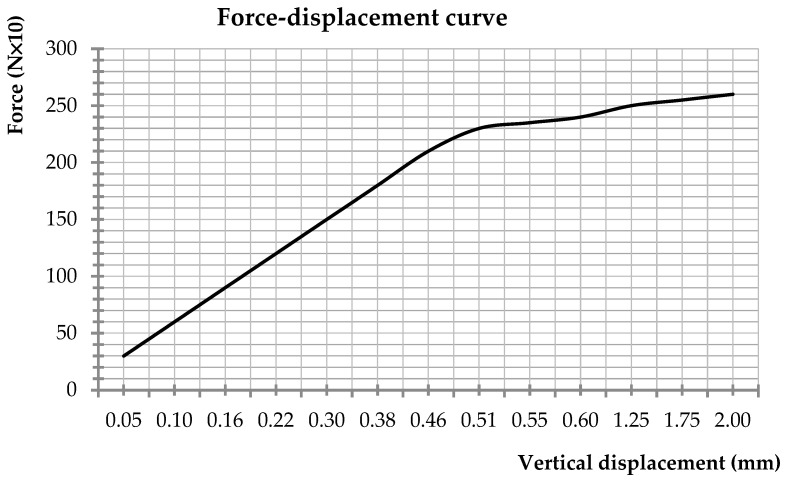
Model 1, force–displacement curve.

Model beams 1 and 2 (classical reinforced concrete) were subjected to a bending moment. Thus, model beam 1′s subjection to a bending moment (classical reinforced concrete) and the values corresponding to the input force and the deformation are shown in Figure 23 and Figure 24.

During the subjection to the force P_model,1_ = 2.35 kN, the longitudinal reinforcement started to yield, this value being comparable to the failure force established by the similitude criteria P_model,M_ = 2.37 kN.

The first cracks appeared in the beam at the value P_model,1,cracks_ = 2.55 kN, and the reinforcement failed at P_model,1,ult_ = 2.80 kN.

The experiment was repeated, this time using the model 2 beam, and the obtained values were P_model,2_ = 2.37 kN—the value at which the reinforcement started to yield; P_model,2,cracks_ = 2.59 kN—the value at which the first cracks appeared; and P_model,2,ult_ = 2.83 kN—the value at which the reinforcement failed.

**Figure 25:**
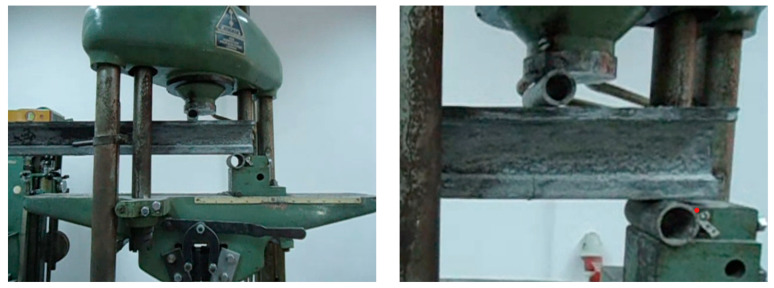
Model 3, shear test.

Models 3 and 4 (classical reinforced concrete) were subjected to shear force (Figure 25), and due to this process, the failure occurred by cracking at the values P_model,3,craks_ = 3.40 kN and P_model,4,cracks_ = 3.34 kN. Both values are in good accordance with the value obtained using the scaling factor on the prototype’s beam value, P_model,V_ = 4.06 kN.

All presented values were established with the objective of estimating, on the scaled-down models, the forces corresponding to the ultimate carrying capacity of the concrete beam. One can assume that the level of the corresponding forces for the serviceability limit state (SLS) is smaller, but further analyses are necessary to determine if the obtained and presented scaling coefficients in the paper lead to values of the forces describing the SLS. It would be interesting to find out if, by directly dividing the obtained values of scaling coefficients by the safety coefficient value, which in this case is 1.35, the correct values of the scaling coefficients for the SLS would be obtained.

## 4. Discussion

The obtained results for both the bending and shear tests on the scaled-down models revealed the usefulness of such types of analyses for estimating the behavior of real structural elements.

The choice of the variables that describe the phenomena, as shown in Table 1, was appropriate because the scaling criteria of the applied input force could be developed as a function of known variables, both in the prototype’s and in the model’s case.

The input’s force scaling factor foresaw the failure force’s value in bending as P_model,M_ = 2.37 kN. The failure of the model in bending occurred at values between 2.35 and 2.40 kN. For the shear test, the failure occurred by cracking at the values P_model,3,craks_ = 3.40 kN and P_model,4,cracks_ = 3.34 kN. The value obtained using the scaling factor on the prototype’s beam value was P_model,V_ = 4.06 kN. Noticing the small difference between the values, one can confirm that the choice of the scaling criteria was conclusive.

From analyzing the comparison between the scaled-down model’s (1:10) behavior and the behavior of the real bridge beam (prototype), it can be noticed that the computations used allow for the simulation of real bridge beam behavior by testing a similar scaled-down model beam and using the scaling factors according to the similitude theory.

The manufacturing of scaled-down concrete elements emphasizes the advantages of reduced manufacturing and testing costs compared to the costs necessary to build and test real-scale elements, the ease of fabrication and laboratory handling, and relatively small values of test forces. It is thus possible to optimize the necessary material consumption for achieving the structural elements of bridges, for estimating their behavior under different loads, and the correct management of the possible risks that may occur, with positive effects on the cost–benefit ratio.

With the occasion of these performed tests, other aspects of the real phenomena could be analyzed. Thus, we studied, based on the same similitude theory and dimensional analysis, the possibility of establishing the ultimate force of a retrofitted beam bridge by using carbon fibers. The tested beams were repaired by matting the existing cracks with special mortars and by the aid of carbon fibers. The repaired models were re-tested in the same conditions as presented in this paper, and the values of the resulting ultimate loads were larger with respect to those obtained on the initial models. Thus, for the same value of the beam sag corresponding to the failure load of P_model,M_ = 2.37 kN on the initial model, the value of the force on the retrofitted model was P_retroff,M_ = 4.30 kN. However, further analyses are necessary for testing other retrofitted scaled-down models to confirm the conclusions of this study.

Moreover, the effect of reinforcement corrosion will also be studied. In the first stage, the reinforcing bars will be immersed in a special solution for a certain time and then used inside the scaled-down model. Subsequently, we will test the model to obtain the values of the forces corresponding to the ultimate state and compare them with those presented in this paper.

## 5. Conclusions

In this paper, the results of utilizing similitude theory in conjunction with dimensional analysis to estimate the ultimate carrying capacity of a typical precast concrete bridge beam are presented. The obtained results align very well with those resulting from calculations based on finite element models considering the typical external actions on the bridge. The findings of this study are encouraging, and further research will be conducted on retrofitted scaled-down models.

Based on the comprehensive analysis presented above, the application of similitude theory and dimensional analysis proves to be a valuable approach for estimating the ultimate carrying capacity of precast concrete bridge beams. The selected variables for the scaling criteria, as outlined in Table 1, appear to be well chosen, allowing for the development of input force scaling factors as functions of known variables in both the prototype and model cases.

The calculated scaling factor for the applied input force successfully predicted a failure force (P_model,M_) of 2.37 kN in bending, closely aligned with the observed failure range of 2.35 to 2.40 kN during the bending test. Similarly, for the shear test, the failure occurred at cracking values of P_model,3,cracks_ = 3.40 kN and P_model,4,cracks_ = 3.34 kN, with the scaling factor yielding a value of P_model,V_ = 4.06 kN. The minimal difference between the predicted and observed values underscores the effectiveness of the chosen scaling criteria.

The comparison between the behavior of the scaled-down model (1:10) and the real bridge beam (prototype) reveals that the applied computations and testing procedures allow for the simulation of real bridge beam behavior. This approach, coupled with the advantages of reduced manufacturing and testing costs, ease of fabrication, and manageable test forces, presents an optimized method for estimating the behavior of structural elements in bridge design.

Furthermore, this study suggests potential applications of similitude theory in analyzing other aspects of the real phenomena, such as retrofitting beams with carbon fibers. Initial tests on retrofitted models indicated an increased ultimate load capacity, offering potential benefits in terms of structural optimization and risk management.

As the research progresses, future investigations will explore additional factors, including the effects of reinforcement corrosion, to further refine and expand the applicability of similitude theory in structural engineering. Overall, the results presented in this paper, corroborated by finite element models, demonstrate the promising potential of similitude theory for predicting the behavior of scaled-down models in the realm of bridge engineering.

## Figures and Tables

**Table 1:** Description of variables and measurement units.

Physical Quantity	Symbol	Unit in S.I.
Weight of the beam	G	kg
Force (input)	P	N (kg m s^−2^)
Yielding strength of the reinforcement	f_yk_	N/mm^2^ (kg m^−1^ s^−2^)
Bending moment	M	N × m (kg m^2^ s^−2^)
Reinforcement area	A_s_	m^2^
Height of the beam	h	m
Width of the beam	b	m
Span	L	m

**Table 2:** Dimensional matrix.

Exponent	Variables							
	G	P	f_yk_	M	As	h	b	L
of kg	1	1	1	1	0	0	0	0
of m	0	1	−1	2	2	1	1	1
of s	0	−2	−2	−2	0	0	0	0

**Table 3:** Necessary materials for obtaining one cubic meter of concrete (1 m^3^).

Material	Amount (kg)	
Cement	550	
Water	242	
Aggregate	1656	Percent(%)
Aggregate dimension 2/4	579.6	35%
Aggregate dimension 1/2	331.2	20%
Aggregate dimension 0.5/1	331.2	20%
Aggregate dimension 0.25/0.5	248.4	15%
Aggregate dimension 0/0.25	165.6	10%

**Table 4:** Compression resistance of the cubes.

	Cube 1	Cube 2	Cube 3
Compression resistance (f_ck,cub_) N/mm^2^	53	52	55
Average compression resistance (f_ck,cub_) N/mm^2^	53.3		

**Table 5:** Tensile resistance of the steels used for the reinforcement.

	Threaded Rod	Smooth Wire
Tensile resistance f_t_ (N/mm^2^)	430	375
Yielding limit f_yk_ (N/mm^2^)	350	300

## Data Availability

Data is unavailable due to privacy or ethical restrictions.
